# Supplementary material for: Differential Patterns of Gut and Oral Microbiomes in Hispanic Individuals with Cognitive Impairment
Source: Microorganisms. 2025 Oct 14;13(10):2350. doi: 10.3390/microorganisms13102350 (PMC12566222; doi:10.3390/microorganisms13102350)
Supplement: Supplementary file 1 [file microorganisms-13-02350-s001.zip › microorganisms-3851373-supplementary.pdf]

Differential Patterns of Gut and Oral Microbiomes in Hispanic Individuals with Cognitive Impairment

Yannick N. Wadop[1], Erin L. Vasquez[1], Julia J. Mathews[1], Jazmyn A. S. Muhammad[1], Rosa Pirela Mavarez[2], Claudia L. Satizabal[1,5,7], Mitzi M Gonzales[1,3], Jeremy Tanner[1], Gladys Maestre[2], Alfred N. Fonteh[4], Sudha Seshadri[1,5,6,7], Tiffany F. Kautz[1,*], Bernard Fongang[1,8,9,#,*]

1. Participants' oral and gut microbial contents differ between the cognitively impaired and normal groups

		Positive correlation	Negative correlation
**Lipids**	**Serum cholesterol**	Prevotella, Eikenella, Lautropia, Filifactor, Megasphaera, Bifidobacterium, Catonella, Peptococcus, Mycoplasma, Phocaeicola	Butyrivibrio, Centipeda, Aggregatibacter, Eubacterium nodatum group, Eubacterium brachy group
	**HDL cholesterol**	Catonella, Lautropia, Aggregatibacter, Mycoplasma	Megasphaera, Solobacterium, Eubacterium nodatum group, Eubacterium brachy group, Shuttleworthia, Bifidobacterium, Fretibacterium
	**LDL cholesterol**	Eikenella, Prevotella, Megasphaera, Bifidobacterium, Lautropia, Filifactor, Catonella, Shuttleworthia, Peptococcus, Mycoplasma, Phocaeicola	Bacteroides, Butyrivibrio, Fretibacterium, Aggregatibacter, Centipeda
	**Triglycerides**	Prevotella, Eubacterium nodatum group, Centipeda, Catonella, Solobacterium, Mycoplasma, Abiotrophia, Aggregatibacter, Phocaeicola, Megasphaera	
**Inflammation**	**C-reactive protein (CRP)**	Butyrivibrio, Solobacterium, Fretibacterium, Megasphaera, Shuttleworthia, Mycoplasma, Centipeda,	Eikenella, Bifidobacterium, Aggregatibacter, Bacteroides, Lautropia, Filifactor
**Diabetes**	**hbA1c**	Prevotella, Centipeda, Shuttleworthia, Eubacterium brachy group, Megasphaera	Eubacterium nodatum group, Eikenella, Lautropia, Catonella, Bacteroides, Peptococcus
	**Glucose**	Prevotella, Solobacterium, Centipeda, Shuttleworthia, Eubacterium brachy group, Mycoplasma, Megasphaera, Phocaeicola	Eubacterium nodatum group, Catonella, Aggregatibacter, Butyrivibrio, Lautropia,
**Oxidation**	**Homocysteine**	Eubacterium nodatum group, Bacteroides	Prevotella, Lautropia, Aggregatibacter, Centipeda, Filifactor, Mycoplasma, Peptococcus

Supplementary Table S1. Oral bacterial genera showing positive or negative correlation with glucose, lipids, CRP, hbA1c, and oxidation.

2. Differential Abundance analysis of the Gut Microbiome and Saliva Microbiome in CI and NC patients

			Increased abundance	Decreased abundance
**Cognition CI vs NC**	**Stool**	Phylum		*Firmicutes*
		Class		*Clostridia*
		Order		*Oscillospirales, Erysipelotrichales*
		Family		*Erysipelatoclostridiaceae, [Eubacterium] coprostanoligenes group, Oscillospiraceae*
		Genus		*[Eubacterium] brachy group, Family XIII UCG-001, Holdemania, Shuttleworthia, Subdoligranulum, UCG-005*
	**Saliva**	Phylum	*Synergistota, Fusobacteriota*	
		Class	*Synergistia, Fusobacteriia*	
		Order	*Synergistales, Mycoplasmatales, Clostridia UCG-014, Fusobacteriales*	
		Family	*Synergistaceae, Mycoplasmataceae*	
		Genus	*Dialister, Fretibacterium, Mycoplasma*	

Supplementary Table S2. Summary of differential oral and gut taxa between CI and NC patients.

3. Enhancing the functional potential of the gut and oral microbial communities associated with cognitive health using KEGG pathways

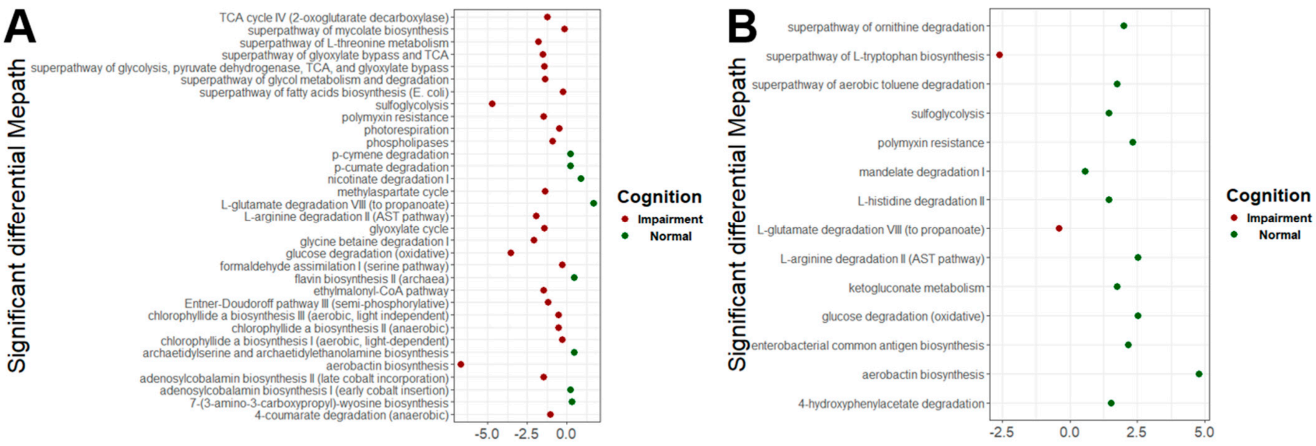

Supplementary Figure S1. Predicted MePath associated with cognition, comparing the Normal and Impairment groups for gut microbiome (A) and oral microbiome (B).

		Increased level	Decreased level
ECs	Oral	CoA-disulfide reductase; Ethanolaminephosphotransferase	Catechol 1,2-dioxygenase;D-arabinitol 4-dehydrogenase;Ferric-chelate reductase (NADPH); Tryptophan 7-halogenase; Acyl-homoserine-lactone synthase; Vanillin synthase; Trans-feruloyl-CoA hydratase; Aerobactin synthase; N(2)-citryl-N(6)-acetyl-N(6)-hydroxylysine synthase; N(6)-hydroxylysine O-acetyltransferase; Oligogalacturonide lyase; Formate dehydrogenase-N; Mannosyl-3-phosphoglycerate phosphatase; NAD(P)(+) transhydrogenase (Si-specific); Streptomycin 3-kinase; Lipid IV(A) 4-amino-4-deoxy-L-arabinosyltransferase; dTDP-4-amino-4,6-dideoxy-D-galactose acyltransferase; 1,6-dihydroxycyclohexa-2,4-diene-1-carboxylate dehydrogenase; Benzoate 1,2-dioxygenase; Sulfolactaldehyde 3-reductase; Aliphatic aldoxime dehydratase;L-lysine N(6)-monooxygenase (NADPH); 23S rRNA (guanine(1835)-N(2))-methyltransferase; Acireductone dioxygenase (Ni(2+)-requiring); Acireductone dioxygenase (Fe(2+)-requiring); Pectin lyase; TDP-N-acetylfucosamine:lipid II N-acetylfucosaminyltransferase; Formimidoylglutamate deiminase
	Gut	4-aminobutyrate--pyruvate transaminase; Malyl-CoA lyase;(2S)-methylsuccinyl-CoA dehydrogenase;5-aminolevulinate synthase;Malonyl-CoA decarboxylase; Dimethylglycine dehydrogenase; NAD(+)--dinitrogen-reductase ADP-D-ribosyltransferase; Methylamine dehydrogenase (amicyanin); Succinyl-CoA--L-malate CoA-transferase; Precorrin-6A synthase (deacetylating); 3-methylfumaryl-CoA hydratase; Demethylspheroidene O-methyltransferase; 1-hydroxycarotenoid 3,4-desaturase; Alanine--glyoxylate transaminase; Serine--glyoxylate transaminase; Serine--pyruvate transaminase; 4-methylaminobutanoate oxidase (formaldehyde-forming); Salicylate 1-monooxygenase; 4-hydroxybenzoate--CoA ligase; Gluconate/galactonate dehydratase; Dimethyl sulfide:cytochrome c2 reductase; Dimethyl sulfide:cytochrome c2 reductase; Pyridoxamine--pyruvate transaminase; 2-methylfumaryl-CoA isomerase;	Spermidine dehydrogenase; GalNAc(5)-diNAcBac-PP-undecaprenol beta-1,3-glucosyltransferase; Acetylserotonin O-methyltransferase; Nicotinate dehydrogenase (cytochrome); Non-reducing end beta-L-arabinofuranosidase; 15,16-dihydrobiliverdin:ferredoxin oxidoreductase; dCTP diphosphatase

Supplementary Table S3. Top 30 differential oral and gut ECs between CI and NC patients.

		Increased level	Decreased level
KOs	Oral	K16819	K02465, K08087, K19734, K09916, K18555, K03381, K18838, K07349, K03225, K00007, K03226, K03230, K03222, K03228, K03219, K03229, K14744, K16324, K03812, K07350, K07310, K11911, K16090, K12290, K09016, K10972, K03220, K07348, K10973
	Gut	K18588, K07716, K14981, K14980, K07234, K13587, K13583, K00410, K13588, K17662, K16871, K13584, K09991, K09987, K18587, K08691, K14448, K06602, K00643, K14447, K01578, K15721, K02132, K00315, K05951, K13013, K13598, K18989, K18990, K07167	

Supplementary Table S4. Top 30 differential oral and gut KOs between CI and NC patients.

3.1 Stool samples

ID	Description	BH p.adjust	KOs ID	count
ko00195	Photosynthesis	7.703e-22	K02705, K02639, K02720, K02703, K02700, K02643, K02718, K02714, K02640, K02711, K02722, K02723, K02717, K02697, K02634, K02694, K02704, K02707, K02709, K02716, K08902, K08903, K02724, K02638, K02693, K02698, K02706, K08906, K02641	29
ko00196	Photosynthesis - antenna proteins	8.927e-17	K02290, K05378, K05379, K05381, K05383, K05384, K05386, K05385, K05377, K05382, K05376, K02092, K02094, K02096, K02097, K02288, K02289, K02093, K02095, K02284, K02285	21
ko03070	Bacterial secretion system	4.318e-9	K02465, K11915, K11016, K11017, K02461, K02462, K02460, K02458, K02459, K02457, K11912, K02452, K03223, K03229, K13301, K04059, K03221, K03425, K03226	19
ko02026	Biofilm formation - Escherichia coli	6.419e-7	K04335, K04333, K04336, K12687, K04334, K07782, K07677, K07678, K07676, K02425, K07689, K02403, K02402, K14051, K07687	15
ko01503	Cationic antimicrobial peptide (CAMP) resistance	7.544e-7	K19079, K13632, K19238, K07264, K07660, K07806, K07637, K12963, K12973, K07771, K13014, K12975, K19236, K07643	14
ko00680	Methane metabolism	2.685e-6	K08691, K00830, K01086, K13942, K00400, K13812, K00442, K00440, K00319, K00443, K00578, K00579, K00580, K00581, K00582, K00583, K00204, K07811, K10978, K01070, K03533, K03532, K18277, K00399, K13039, K06034	26
ko01200	Carbon metabolism	0.000151	K08691, K14448, K14447, K09709, K14472, K00830, K01086, K14471, K05308, K01602, K14470, K07511, K15918, K13942, K13812, K00319, K00578, K00579, K00580, K00581, K00582, K00583, K00204, K00116, K01637, K01690, K01638, K01782, K01070, K01682, K01825, K00399, K00242, K19243	34
ko02040	Flagellar assembly	0.000248	K02394, K02393, K02386, K02399, K02391, K02423, K10941, K02425, K02403, K02402, K03516	11
ko04112	Cell cycle - Caulobacter	0.000514	K07716, K13587, K13583, K13588, K13584, K13589, K06985, K13582	8
ko00860	Porphyrin metabolism	0.00116	K00643, K02228, K11336, K04037, K04039, K05369, K00214, K05370, K05371, K10960, K04040, K04038, K03428, K03403, K13543, K07215, K00228	17
ko00984	Steroid degradation	0.00409	K15981, K03333, K16049, K16048, K05898	5
ko00630	Glyoxylate and dicarboxylate metabolism	0.00450	K08691, K14448, K14447, K01432, K00830, K01602, K15918, K01637, K01638, K01682, K12972, K03418, K00090	13
ko00190	Oxidative phosphorylation	0.00451	K00410, K02132, K05575, K05577, K05586, K05588, K05578, K05572, K05573, K05581, K05583, K05584, K05585, K05582, K05579, K05574, K00242, K02297, K02300, K02298, K02299	21
ko00364	Fluorobenzoate degradation	0.00829	K01061, K05783, K05549, K05550, K05784	5
ko00540	Lipopolysaccharide biosynthesis	0.0106	K02848, K03275, K03276, K12981, K12985, K07264, K11211, K12973, K12975	9
ko00740	Riboflavin metabolism	0.0158	K08096, K14653, K19286, K03788, K01497, K05368, K12152, K00299	8
ko00640	Propanoate metabolism	0.0199	K01578, K07511, K11264, K00932, K01782, K01682, K01825, K17489, K19745, K13923, K01908	11
ko00622	Xylene degradation	0.0219	K10622, K05783, K05549, K05550, K10621, K05784	6
ko00410	beta-Alanine metabolism	0.0219	K01578, K00316, K07511, K13799, K01782, K01825, K00276	7
ko00920	Sulfur metabolism	0.0220	K17225, K17285, K16964, K16965, K16966, K00988, K08353, K08354, K00381, K00299, K03119, K04091	12
ko00130	Ubiquinone and other terpenoid-quinone biosynthesis	0.0220	K18534, K12073, K18286, K03181, K18800, K19222, K03184, K03185	8
ko00030	Pentose phosphate pathway	0.0266	K01086, K05308, K13812, K01690, K00032, K11441, K05774, K00090, K00117, K19243	10
ko02030	Bacterial chemotaxis	0.0284	K03776, K05876, K12368, K05874, K10108	5
ko00362	Benzoate degradation	0.0290	K04105, K04107, K01782, K01825, K10622, K05783, K05549, K05550, K14727, K10621, K05784	11
ko00260	Glycine, serine and threonine metabolism	0.0345	K00643, K00315, K15783, K15786, K15784, K00830, K15918, K00276, K12525, K12972, K00090	11
ko00760	Nicotinate and nicotinamide metabolism	0.0385	K19191, K18028, K13995, K15357, K14974, K18030, K18029, K08324, K00322, K11751	10
ko00240	Pyrimidine metabolism	0.0385	K16904, K13799, K09023, K09021, K09018, K08723, K09024, K09019, K08320, K09913, K11751	11
ko00660	C5-Branched dibasic acid metabolism	0.0385	K08691, K18288, K14472, K14471, K18289	5

Supplementary Table S5. Summary of differential gut KOs grouped in their KEGG pathways.

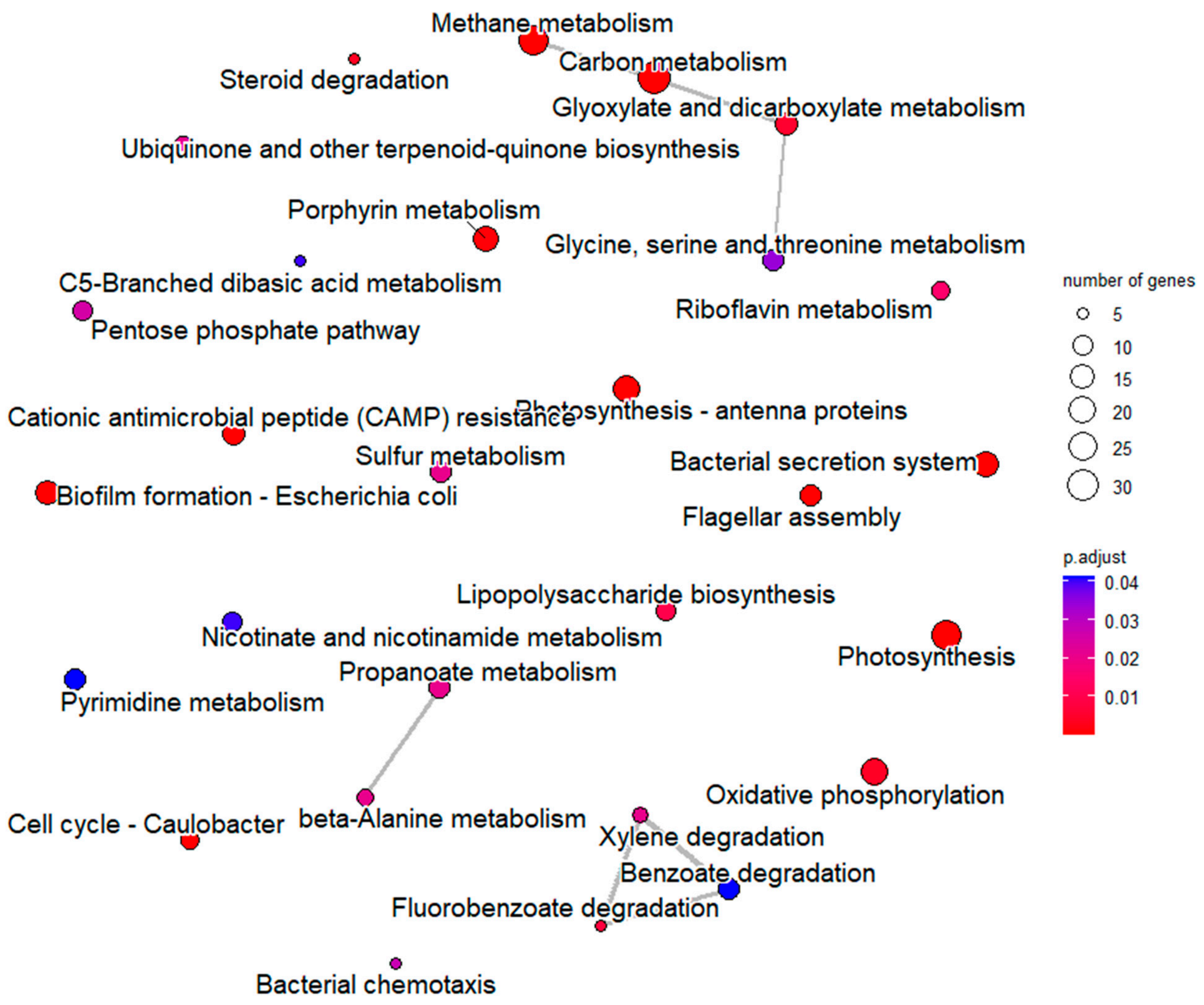

Supplementary Figure S2. Emap plot of enriched KEGG pathways illustrating their network association for gut microbiome.

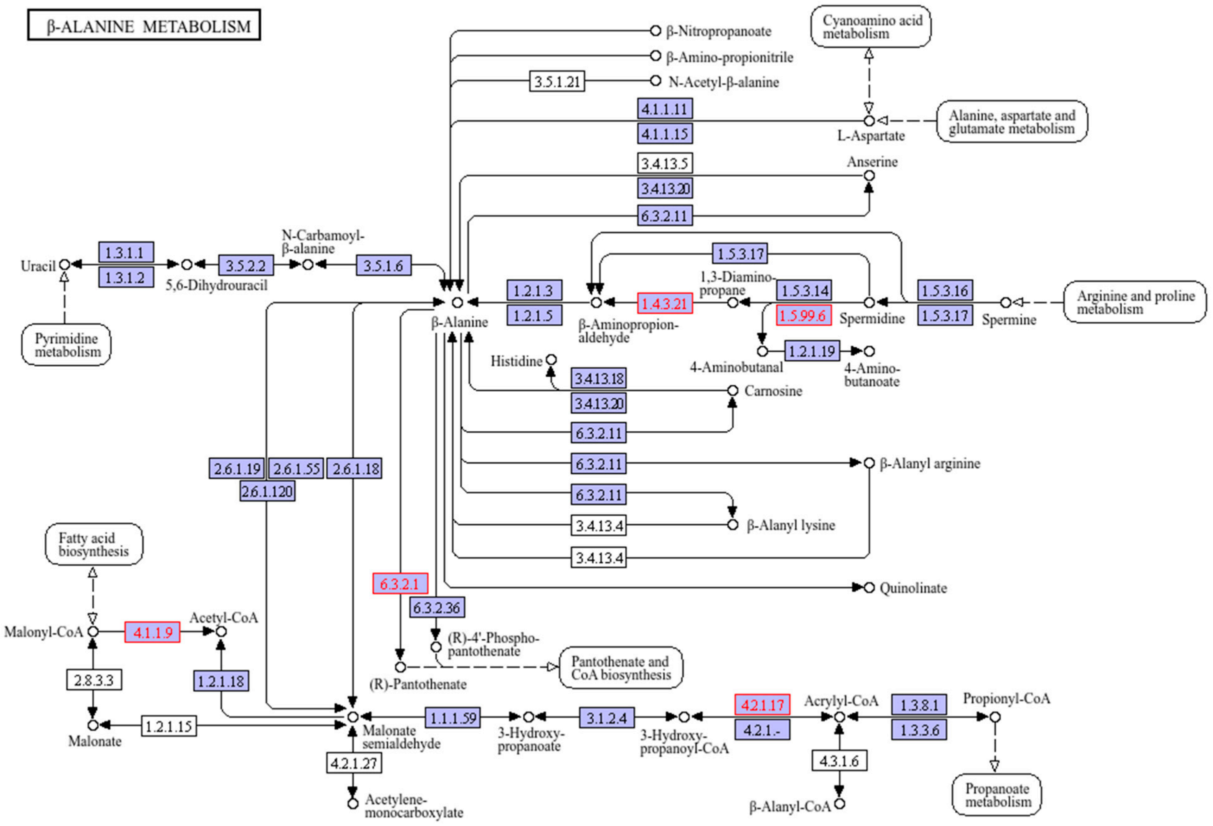

Supplementary Figure S3. KEGG metabolic map highlighting the KOs (red color) that were identified in *beta-Alanine metabolism*.

3.2 Saliva samples

ID	Description	BH p.adjust	KOs ID	count
ko03070	Bacterial secretion system	3.359e-12	K02465, K03225, K03226, K03230, K03222, K03228, K03219, K03229, K03221, K02452, K02459, K11016, K02460, K02464, K02458, K02462, K02457, K02461	18
ko01503	Cationic antimicrobial peptide (CAMP) resistance	7.815e-9	K19238, K19077, K19080, K07264, K07806, K01406, K07771, K10011, K12963, K07660, K12975, K13014, K12973	13
ko00330	Arginine and proline metabolism	0.00105	K00137, K05526, K09472, K12252, K06447, K00673, K01484, K1225, K12254, K00840, K09473	11
ko00364	Fluorobenzoate degradation	0.00105	K03381, K05783, K05550, K05549, K01721	5
ko00440	Phosphonate and phosphinate metabolism	0.00114	K00993, K06165, K06164, K05780, K06166, K06163, K06162	7
ko00860	Porphyrin metabolism	0.001602	K02225, K13543, K03394, K05895, K02304, K06042, K02189, K05936, K05934, K02191, K03399, K13542	12
ko00627	Aminobenzoate degradation	0.00274	K11311, K05599, K10219, K18541, K05600, K16320, K16319, K01865, K01721	9

ko00907	Pinene, camphor and geraniol degradation	0.00284	K13776, K01782, K13774, K13779, K01825	5
ko00051	Fructose and mannose metabolism	0.00284	K00007, K07026, K18334, K18335, K19270, K19291, K19290, K01795, K07046, K00045	10
ko02026	Biofilm formation - Escherichia coli	0.00469	K03567, K04333, K07689, K07677, K18968, K14051, K07687	7
ko00350	Tyrosine metabolism	0.00547	K13951, K13574, K01826, K05921, K00146, K10219, K00483, K00455	8
ko00362	Benzoate degradation	0.00547	K03381, K05783, K05550, K05549, K07823, K10219, K01782, K01825, K01055	9
ko02025	Biofilm formation - Pseudomonas aeruginosa	0.00748	K13060, K13489, K13491, K11444, K07689, K18100, K19291, K10941	8
ko00071	Fatty acid degradation	0.0145	K13951, K06445, K00529, K00255, K01782, K01825	6
ko01240	Biosynthesis of cofactors	0.0145	K02225, K03184, K13543, K18800, K03394, K05895, K02304, K19222, K01432, K06042, K02189, K05936, K05934, K02191, K03399, K13940, K13542, K06989	18
ko01220	Degradation of aromatic compounds	0.0227	K03381, K05783, K05550, K05549, K01826, K00529, K05921, K10219, K00483, K00455, K10676, K01055	12
ko00543	Exopolysaccharide biosynthesis	0.0371	K12582, K16701, K02852, K03819, K19291, K19290	6

Supplementary Table S6. Summary of differential oral KOs grouped in their KEGG pathways.

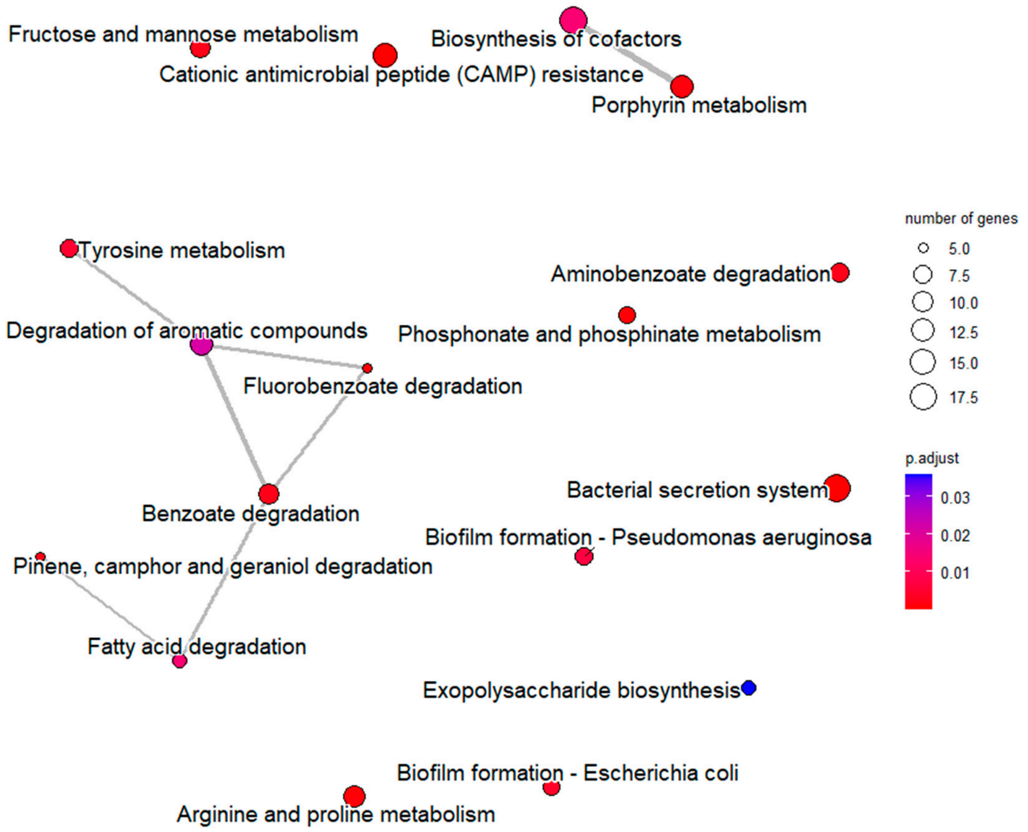

Supplementary Figure S4. Emap plot of enriched KEGG pathways illustrating their network association for oral microbiome.

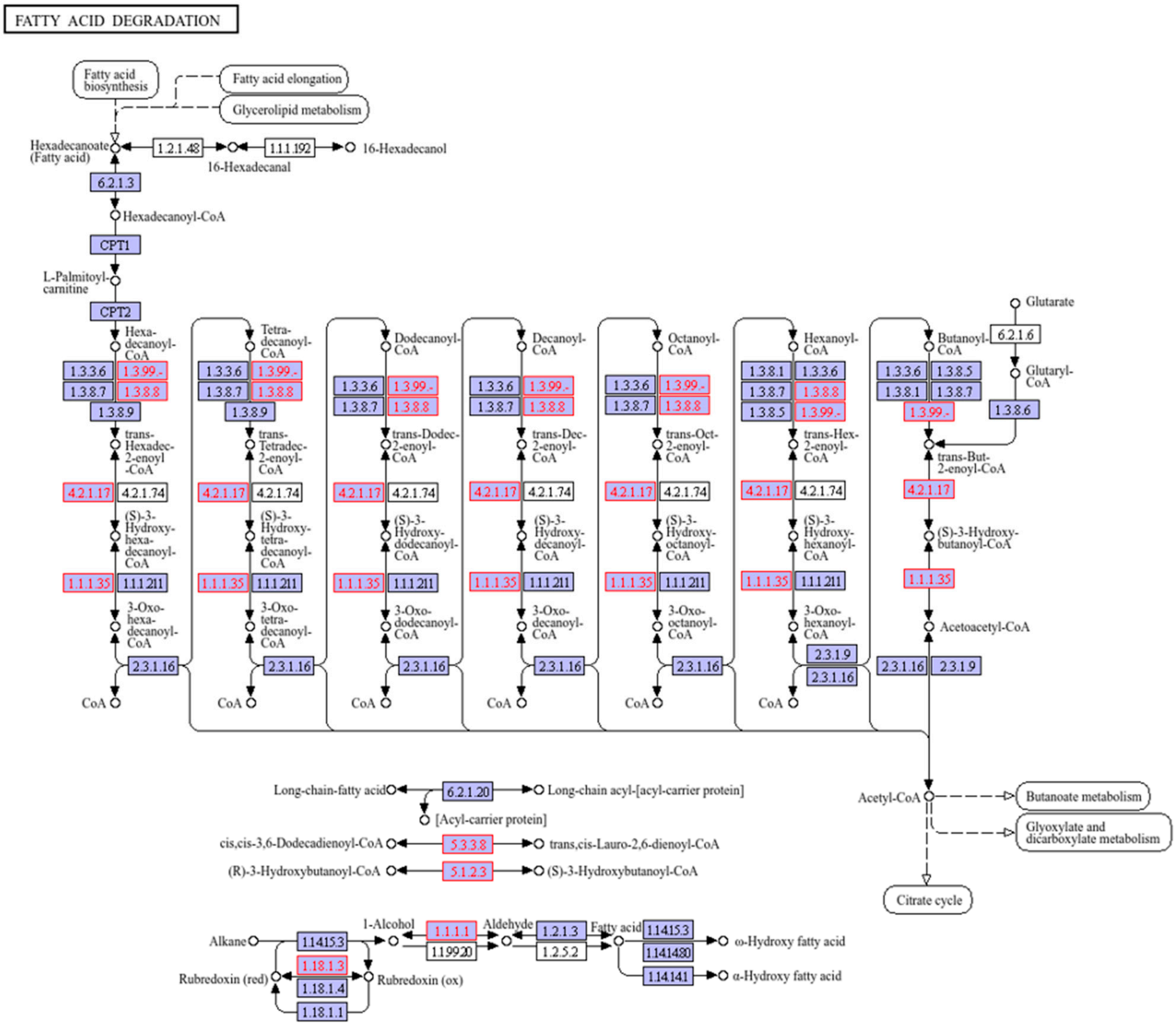

Supplementary Figure S5. KEGG metabolic map highlighting the KOs (red color) that were identified in *Fatty acid degradation*.
